# Genome-wide association analysis reveals the function of *DgSAUR71* in plant height improvement

**DOI:** 10.1186/s12870-025-06246-x

**Published:** 2025-02-22

**Authors:** Xiaoheng Xu, Guangyan Feng, Peng Li, Shuai Yu, Feixiang Hao, Gang Nie, Linkai Huang, Xinquan Zhang

**Affiliations:** College of Grassland Science and Technology, Sichuan Agricultural University, Chengdu, 611130 China

**Keywords:** Plant height, Forage, Orchardgrass, GWAS, *SAUR71*

## Abstract

**Background:**

Orchardgrass (*Dactylis glomerata* L.) is one of the four most economically important forage grasses cultivated globally and serves as an excellent perennial forage with high ecological value. Plant height is a key determinant of both biomass and grain yield. While numerous genes regulating plant height have been identified in annual crops, no such genes have been reported for orchardgrass.

**Results:**

In this study, we analyzed the relationship between plant height and biomass yield in a natural population of 264 orchardgrass genotypes and found that a plant height of 90–110 cm contributed to the maximum biomass yield. Genome-wide association analysis (GWAS) identified 23 candidate loci associated with plant height, corresponding to 62 candidate genes. Among these, *DgSAUR71*, a member of the small auxin-up RNA (SAUR) gene family, emerged as a novel candidate gene associated with plant height. Functional analysis revealed that *DgSAUR71* slightly reduced plant height in rice (*Oryza sativa* L.) and was involved in regulating plant height in orchardgrass.

**Conclusions:**

This study demonstrates that plant height is an important contributor for optimizing biomass yield in orchardgrass, with an optimal range identified. *DgSAUR71* was identified as a gene associated with plant height through GWAS and shown to negatively regulate plant height. These findings provide new insights into plant height regulation in orchardgrass and contribute to advancing crop height diversification research.

**Supplementary Information:**

The online version contains supplementary material available at 10.1186/s12870-025-06246-x.

## Background

Plant height is one of the most important contributors of plant architecture, influencing biomass, yield, and lodging resistance [1]. It is regulated by various phytohormones and genes [2–4]. During the 1960s and 1970s, dwarf varieties with defects in the gibberellin (GA) pathway were extensively utilized to enhance wheat and rice yields globally, an achievement termed the “Green Revolution.” This remains the most successful example of breaking cereal yield potential [5, 6]. Key genes involved in this process, such as *sd1* in rice (*Oryza sativa L.*), *rht-1* in wheat (*Triticum aestivum L.*), and *dwarf 8* in maize (*Zea mays L.*), play crucial roles in controlling plant height through the GA pathway [7–9]. Brassinosteroids (BRs), another class of phytohormones akin to GAs, are also integral to plant height regulation. A defect in either BR biosynthesis or signaling pathways often result in dwarfism, with genes such as *DWF4*, *BRI1*, *BES1* and *BZR1* implicated in these processes [10–12]. Recent studies have identified members of the auxin (IAA) efflux transporter (PIN) family, including *OsPIN2* and *GhPIN3*, as contributors to plant height regulation in species [13, 14]. Strigolactones (SLs), a newer class of phytohormones, also influence plant height, with *CCD7*, a biosynthetic gene in the SL pathway, positively regulating height [15]. Although numerous genes regulating plant height have been identified in crops, only a few have been applied in genetic improvement programs [16–18]. Moreover, research on plant height regulation has predominantly focused on annual crops, such as rice and wheat. To uncover the diverse regulatory mechanisms of plant height across species, further exploration of novel plant height-related genes in different species is needed.

Orchardgrass (*Dactylis glomerata* L.) is among the top four economically significant forage grasses cultivated globally and is a valuable perennial forage with considerable ecological value [19, 20]. It has been widely grown and utilized worldwide due to its high adaptability, nutritional value, and biomass production [21, 22]. In addition, orchardgrass plays a vital role in ecological restoration, as it has been employed to mitigate rocky desertification in southwest China owing to its remarkable tolerance to various abiotic stresses [23–25]. While numerous studies have investigated the effects of abiotic stress and flowering in orchardgrass, other aspects of its biology remain underexplored [26–28]. As a forage, biomass yield has been a primary focus of research, with plant height identified as a key contributor to both biomass and grain yield [1, 18, 29, 30]. Plant height is also an important agronomic trait that exhibits pleiotropic interactions with other traits such as tiller number, biomass, seed yield, and lodging resistance [1, 4, 18, 30, 31]. In southwest China, orchardgrass is typically harvested at the early flowering stage to maximize both biomass and nutritional value [32, 33]. Given the influence of plant height on tiller number and biomass, optimizing plant height is beneficial for maximizing biomass yield. Therefore, regulating plant height offers a promising approach to enhance forage production. However, no genes associated with plant height regulation in orchardgrass have been identified to date.

As an important perennial forage, orchardgrass shares a close genetic relationship with major crops such as rice, barley (*Hordeum vulgare* L.) and wheat [28]. Research on plant height regulatory genes in orchardgrass contributes to broadening the understanding of plant height regulation in major crops like rice and barley. Compared with annual crops, plant height also influences aboveground biomass of orchardgrass, making it essential to explore the regulatory genes involved.

In this study, 23 loci and 62 associated genes regulating plant height in orchardgrass were identified through genome-wide association analysis (GWAS). Among these, *DgSAUR71*, a member of the small auxin-up RNA gene family, was shown to negatively regulate the plant height in rice. Therefore, these results will be beneficial to reveal the regulatory mechanism of plant height in orchardgrass and offer a foundation for identifying target genes for forage breeding in the future.

## Methods

### Plant materials and field experiment design

A natural population comprising 264 genotypes from various regions worldwide was cultivated in Yaan’s fields (29°98′N, 102°97′E, altitude 600 m) and Dayi’s fields (30°62′N, 103°26′E, altitude 1100 m) in China during 2020–2022. Two replicates of each genotype were grown at both locations. These replicates, obtained through vegetative propagation, were randomly distributed in each field. The spacing between plants was 80 cm × 100 cm, and they were grown under the natural condition of the experiment field trial. Before the experiment, a base fertilizer of 225 kg/hm^2^ was applied, with no additional fertilizer used during the study. Both Dayi and Ya ‘an experienced ample rainfall, and no artificial irrigation was provided. The plant height and biomass yield were measured at the early flowering stage, and the average values from the replicates were used for further analysis (Table S1 and S2). The Pearson correlation coefficients between phenotypes across different years and locations were analyzed using the R package corrplot. The correlation analysis of plant height and biomass were performed though Pearson correlation coefficient with R package corrplot. The normal distribution of relevant data was check by IBM SPSS Statistics 27.

### GWAS analysis

The raw sequencing data and SNPs of all orchardgrass genotypes used in the GWAS analysis are publicly available under the accession number PRJCA018363 (https://ngdc.cncb.ac.cn/gsa*)* [34]. SNPs were filtered using VCFtools [35] with the following parameters: depth > 12, miss < 0.5, maf > 0.05, resulting in 90,796 high-quality SNPs retained for subsequent analysis. Phenotypic data for plant height in orchardgrass, collected from two locations over two years, was analyzed using the best linear unbiased prediction (BLUP) method via the R package lme4 [36–38]. These BLUP values were then used for GWAS analysis employing the mixed linear model (MLM) in GEMMA [39]. LD decay was calculated using r^2^ values with a significance threshold of *p* < 0.05, and a 50 kb distance was set as the candidate gene search window (Fig. S1). Significant SNPs were filtered with a threshold of *p* > 10^− 4^ [40]. Manhattan and Q-Q plots were generated using the ggplot2 package in R [41]. These significant SNPs from the GWAS results were mapped to the orchardgrass reference genome, and genes located within 50 kb upstream and downstream of these SNPs were considered candidate genes [42].

### The analysis of candidate genes and their homologous genes

Gene Ontology (GO) and Kyoto Encyclopedia of Genes and Genomes (KEGG) analyses of associated genes were performed using the OmicShare platform [43]. The *SAUR71* protein sequence from *Aegilops tauschii*, *Brachypodium distachyon*, *Hordeum vulgare*, *Sorghum bicolor*, *Zea mays*, *Oryza sativa*, and *Arabidopsis thaliana* were downloaded from NCBI, and their multiple sequence alignment was conducted using SnapGene software [44]. Three-dimensional protein structures were generated via the SWISS-MODEL homology modeling pipeline (https://swissmodel.expasy.org/*)* [45]. Homologous proteins were used for the multiple sequence alignment via ClustalW, and phylogenetic analysis was conducted using the Neighbor-Joining (NJ) method in MEGA (Version 7.0) with 1000 bootstrap replicates and the Poisson model [46].

### The construction and transformation of vectors

The *DgSAUR71* coding sequence from *2006 to 1* was cloned into a 19T vector using the primer pair *DgSAUR71-p1* (Table S3) and subsequently into the binary vector pCAMBIA1300 under the CaMV35S promoter with *DgSAUR71-p2* (Table S3). The recombinant plasmid was introduced into *Agrobacterium tumefaciens* (*strain EHA105*) and used to transform *Oryza sativa* (ZH11) callus, generating five positive T_0_ transgenic lines verified with *DgSAUR71-p3* (Fig. S2). Two homozygous T_3_ lines were used for expression analysis and field phenotypic observation.

### The phenotype and expression analysis in transgenic plants

All transgenic rice lines were grown in the paddy fields of Sichuan Agricultural University, Chengdu, China, under natural conditions during the growing season. In the paddy fields, compound fertilizer (150 kg/hm^2^) was applied before transplanting, and urea fertilizer (100 kg/hm^2^) was applied at the tillering stage. The water level in the paddy fields was maintained at ~ 10 cm. Ten replicates were used for each transgenic line or wildtype in the field experiment. For *DgSAUR71* overexpressed transgenic lines, plant height was measured at flowering, and three replicate samples for expression analysis were collected during this period. These samples were processed for expression analysis by extracting total RNA using the HiPure plant RNA Mini Kit (MAGEN, Guangzhou, China). Subsequently, these RNA samples were reverse transcribed into first-strand cDNA using the MonScript™ RTIII All-in-One Mix with dsDNase (Monad, Wuhan, China) and were then used as input into a qRT-PCR assay. qRT-PCR was performed using MonAmp™ SYBR Green qPCR Mix (Monad, Wuhan, China) with a total reaction volume of 10 µl, consisting of 5.0 µl SYBR Green qPCR Mix, 3.0 µl H_2_O, 0.5 µl of each primer (10 µM), and 1 µl cDNA (4-fold dilution, 200 ng/µl). Gene expression levels were analyzed using the 2 ^−△Ct^ method with *Os-actin* as the internal reference gene [47–50]. Three replicates were performed for each experiment, and all primers were designed using Primer5 (Table S3).

## Results

### The relationship between maximum biomass yield and plant height in orchardgrass

**Fig. 1:**
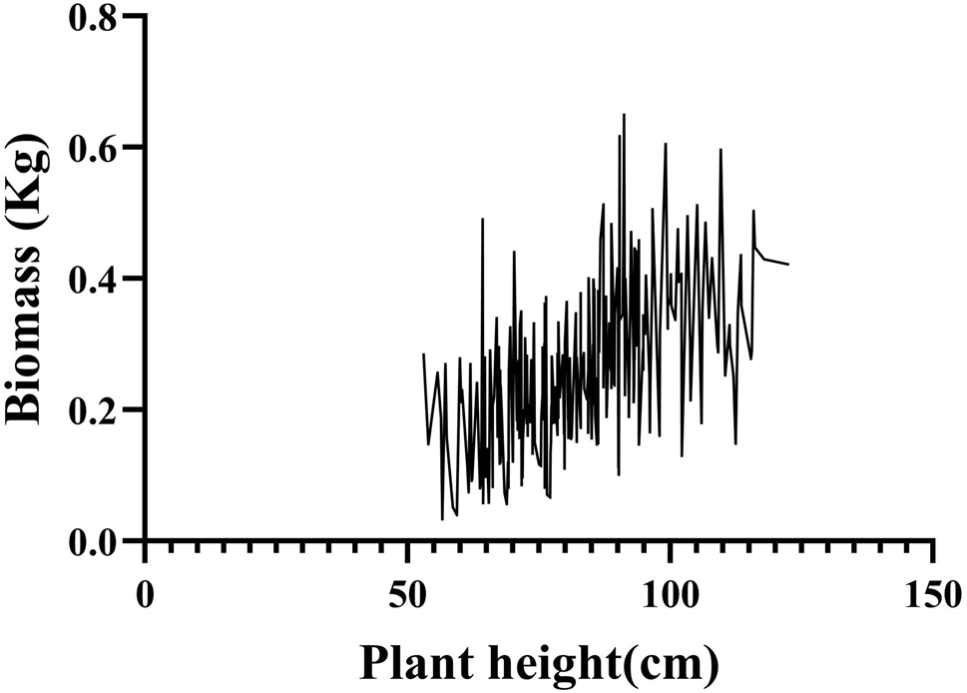
The variation of biomass yield and plant height in orchardgrass GWAS population

To examine the relationship between maximum biomass and plant height in orchardgrass, we analyzed the correlation between plant height and biomass yield in the GWAS population. Plant height was identified as a significant contributor to biomass yield (Fig. S3). As plant height increased, biomass yield per plant also increased (Fig. 1). Biomass reached its maximum value of 0.65 kg when plant height was approximately 90 cm. After this point, biomass yield slightly decreased with further increases in plant height (Fig. 1). Overall, plant heights of 90–110 cm contributed to maximum biomass yield, with the optimal plant height being advantageous for achieving maximum biomass in orchardgrass.

### The frequency distribution analysis of plant height in genome-wide association population

**Fig. 2:**
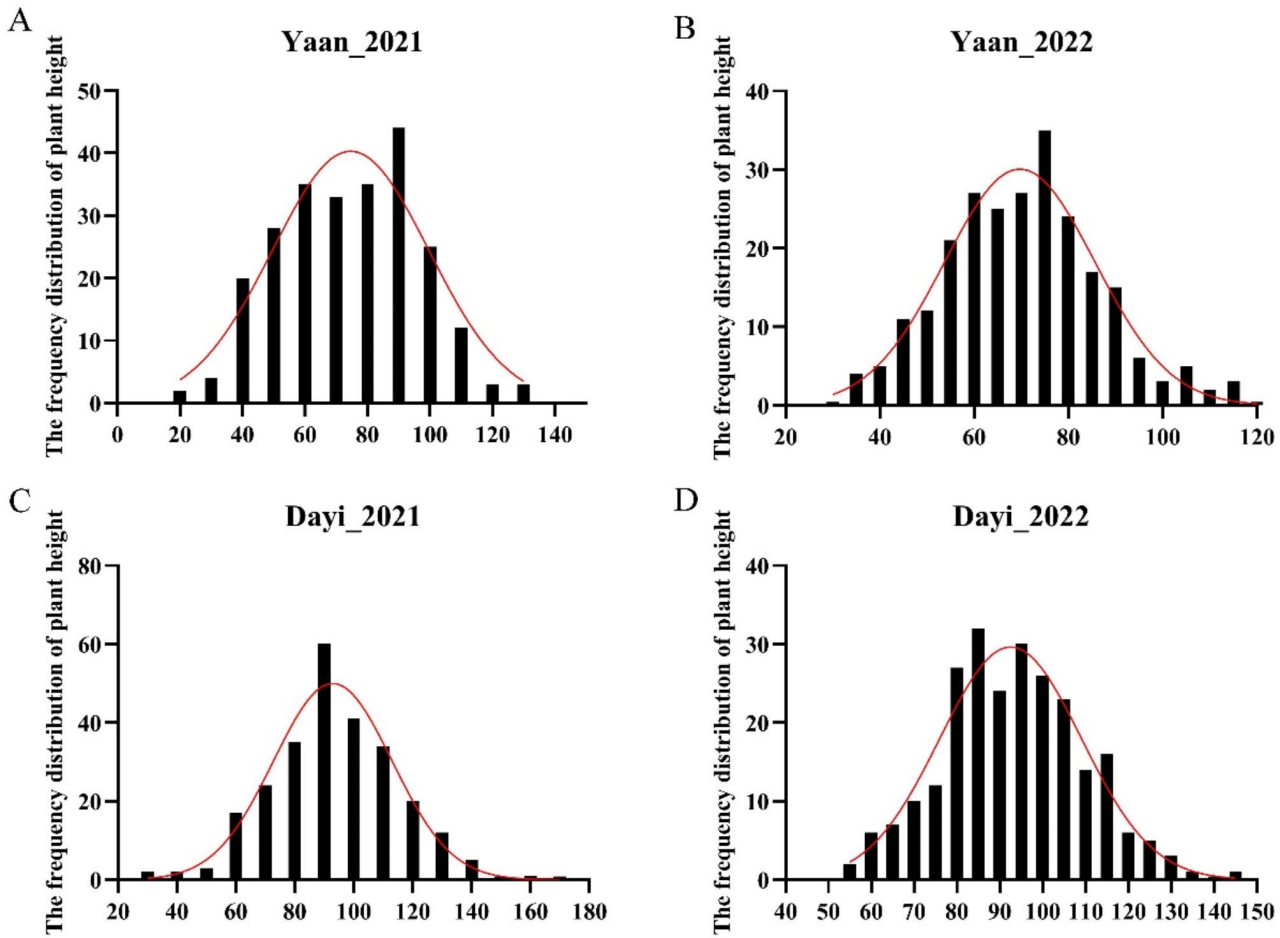
The frequency distribution of plant height in GWAS population in different locations and years. (**A**) Yaan in 2021; (**B**) Yaan in 2022; (**C**) Dayi in 2021; (**D**) Dayi in 2022

To explore candidate genes for plant height in orchardgrass, we first evaluated plant height phenotypic data from the GWAS population. In Yaan_2021, the maximum plant height was 128.37 cm, the minimum was 22.88 cm, and the mean was 74.26 cm (Fig. 2A). In Yaan_2022, the maximum was 116.78 cm, the minimum was 32.80 cm, and the mean was 70.36 cm (Fig. 2B). In Dayi_2021, the maximum was 160.10 cm, the minimum was 31.02 cm, and the mean was 92.91 cm (Fig. 2C). In Dayi_2022, the maximum was 144.75 cm, the minimum was 55.33 cm, and the mean was 93.18 cm (Fig. 2D). In general, mean plant height in Yaan was lower than in Dayi for both 2021 and 2022, with the average plant height in Dayi_2021 closely matching that in Dayi_2022. The phenotypic data from different years and locations were all close to a normal distribution (Fig. 2 and Table S1). Additionally, these phenotypic data between different years and locations were relevant and reproducible (Fig. S4), supporting their use in subsequent analysis.

### Candidate genes of plant height identified by GWAS analysis in orchardgrass

**Fig. 3:**
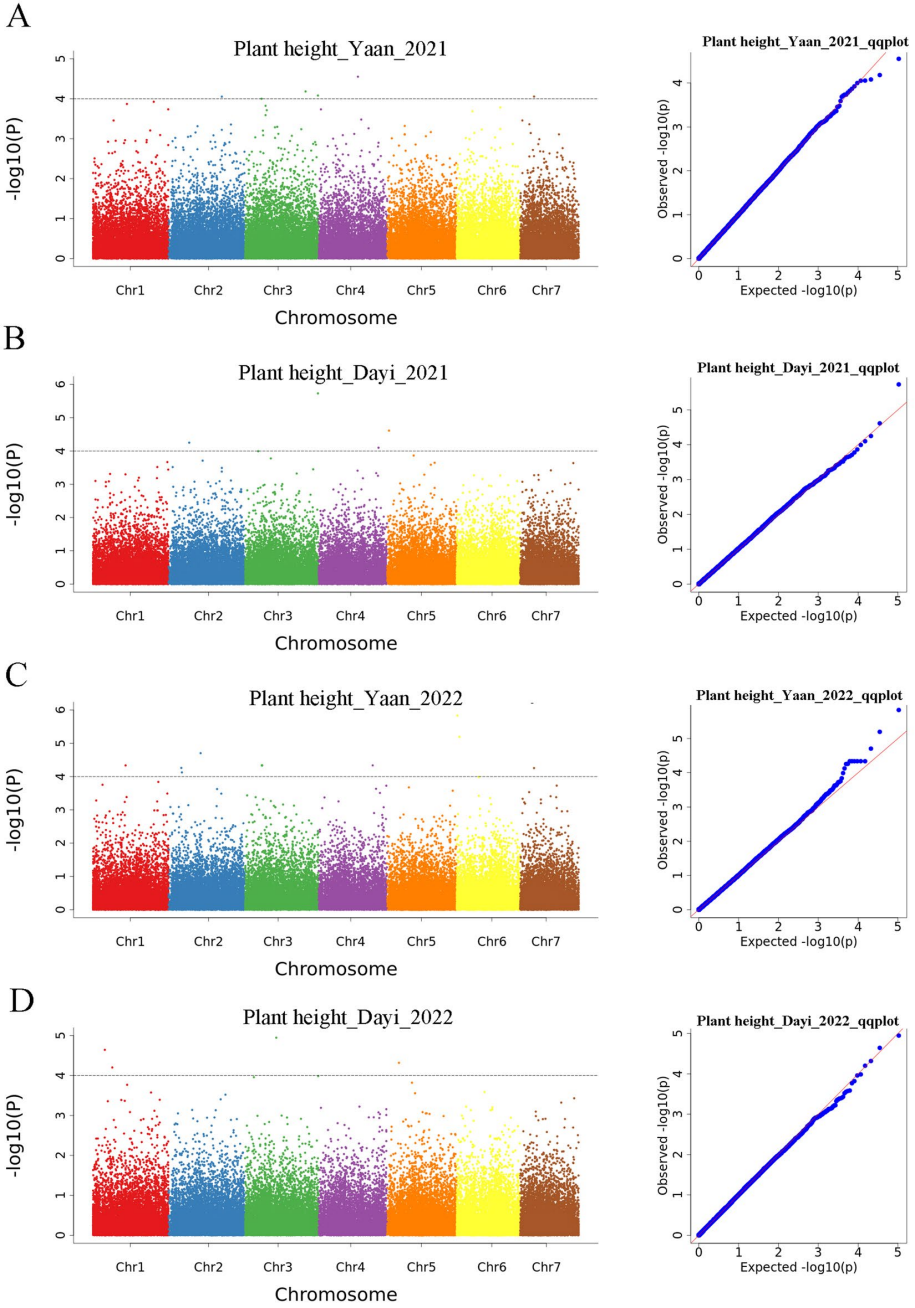
Manhattan plot for GWAS on plant height in (**A**) Yaan_2021; (**B**) Dayi_2021; (**C**) Yaan_2022; and (**D**) Dayi_2022. The dashed black line indicates the threshold − log_10_(*P*) = 4

To identify candidate genes regulating plant height in orchardgrass, we conducted a GWAS analysis using a Mixed Linear Model. We identified six loci exceeding a significant threshold on Yaan_2021 and four loci on Dayi_2021 (Fig. 3A, B). On Yaan_2022, nine loci significantly exceeded the threshold, and four loci on Dayi_2022 did the same (Fig. 3C, D). In total, 23 loci were identified, associated with 62 genes (Table S4). No homologous genes regulating plant height were found among these 62 candidate genes. Most of the associated genes were involved in biological and metabolic processes, with most functioning in binding, according to GO term results (Fig. S5). KEGG analysis showed that six genes were involved in various metabolic pathways, such as glutathione, cysteine, and methionine metabolism (Table S5), and only *DG2C02300* was linked to plant hormone signal transduction and auxin response (Tables S4 and S5). Given that plant height is regulated by GAs and IAA, we selected *DG2C02300*, which is involved in auxin response, for further experiments.

### Identification of the candidate gene through multi-species alignment

**Fig. 4:**
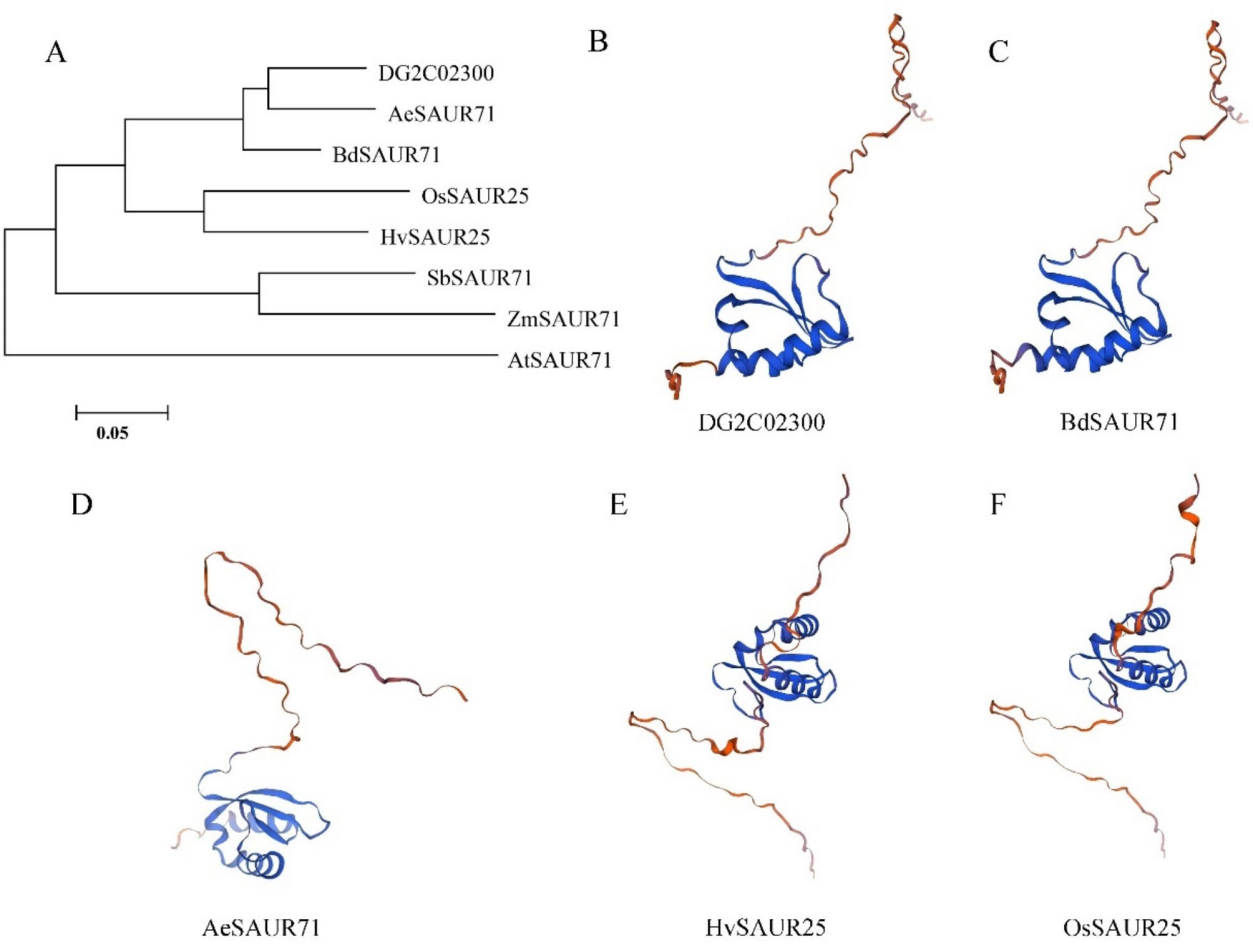
The phylogenetic tree and three-dimensional protein structure of SAUR71 between orchardgrass and its closely related species. (**A**) The phylogenetic tree of SAUR71 in *Aegilops tauschii*, *Brachypodium distachyon*, *Hordeum vulgare*, *Sorghum bicolor*, *Zea mays*, *Oryza sativa*, *Arabidopsis thaliana* and orchardgrass. The prediction of three-dimensional protein structure of (**B**) DG2C02300; (**C**) BdSAUR71; (**D**)AeSAUR71; (**E**) HvSAUR25; (**F**) OsSAUR25

The protein sequence of DG2C02300 in orchardgrass was aligned with other species and found to be homologous to SAUR71 in *Aegilops tauschii*, *Brachypodium distachyon*, *Sorghum bicolor*, and *Zea mays*, or to SAUR25 in *Oryza sativa* and *Hordeum vulgare* (Fig. S6). To explore their genetic relationships, we constructed a phylogenetic tree, which revealed that DG2C02300 was most similar to AeSAUR71 and BdSAUR71, followed by OsSAUR25 and HvSAUR25 (Fig. 4A). The three-dimensional analysis of the predicted protein structure indicated that DG2C02300 was most similar to BdSAUR71, rather than AeSAUR71, OsSAUR25, or HvSAUR25 (Fig. 4B-F). Therefore, the predicted gene *DG2C02300* was named *DgSAUR71*, and it is classified within the small auxin-up RNA gene family.

### *DgSAUR71* decreased plant height in rice

**Fig. 5:**
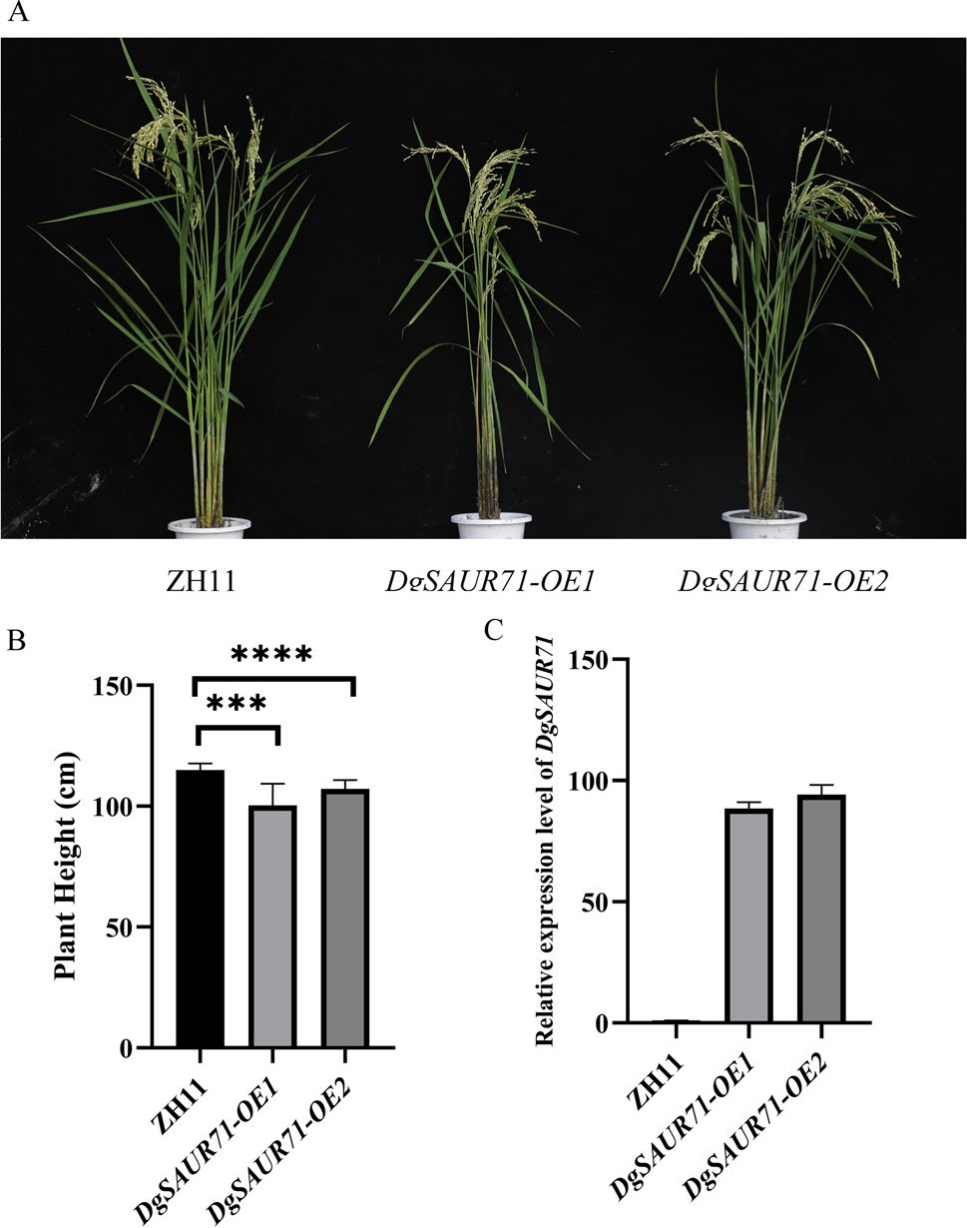
*DgSAUR71* regulates plant height in rice. (**A**) Morphologies of ZH11, *DgSAUR71-OE1* and *DgSAUR71-OE2*. (**B**) The plant height of ZH11, *DgSAUR71-OE1* and *DgSAUR71-OE2*. (**C**) The relative expression levels of *DgSAUR71* in ZH11, *DgSAUR71-OE1* and *DgSAUR71-OE2*. (**B**) and (**C**) are presented as means ± SD, *n* = 10 in (**B**) and *n* = 3 in (**C**). NS, not significant; *P < 0.05, **P < 0.01, ***P < 0.001, ***P < 0.001, Tukey’s multiple comparisons test

To investigate the function of *DgSAUR71*, we overexpressed it in ZH11 (*Oryza sativa*) using the *CaMV35S* promoter. The results showed that the mean plant heights of *DgSAUR71-OE1* and *DgSAUR71-OE2* were 100.3 cm and 107.2 cm, respectively, compared to 114.9 cm in the wildtype (Fig. 5A, B, and Table S6). The plant height of *DgSAUR71-OE1* was 14.6 cm shorter, and that of *DgSAUR71-OE2* was 7.7 cm shorter than the wildtype (Fig. 5A, B). On average, The plant height of lines overexpressing the *DgSAUR71* gene were on average 6.7% shorter than their untransformed control comparator plants. The expression levels of *DgSAUR71* in *DgSAUR71-OE1* and *DgSAUR71-OE2* were significantly higher than in the wildtype (Fig. 5C). Overexpression of *DgSAUR71* slightly reduced plant height in rice, and it was negatively correlated with plant height. Overall, these results suggest that *DgSAUR71* negatively regulates plant height in rice.

## Discussion

Plant height, a key agricultural trait, is linked to various factors such as biomass, yield, and lodging resistance [1]. It plays a crucial role in the yield and profitability of various crops. For instance, optimal plant height aids in manual management and reduces harvesting difficulty in woody horticultural plants, helping to maximize crop yield through either a mechanical or manual process [51, 52]. In cereal crops, plant height influences grain yield and net profit by affecting tiller number, lodging resistance, and mechanization [16, 53]. However, for forage crops, plant height not only impacts tiller number, lodging resistance, and seed yield but also affects above-ground biomass yield [18, 54–57]. While numerous genes regulating plant height in cereals have been identified [17], few genes controlling plant height in forage crops have been discovered. Therefore, the use of plant height genes in forage breeding remains significantly underdeveloped. To improve forage biomass, further exploration and evaluation of new plant height genes for breeding are necessary.

As one of the top four economically important forage grasses globally [19, 20], orchardgrass has been widely cultivated due to its high adaptability, nutritional value, and biomass [21, 22]. The biomass yield in triticale (× *Triticosecale* Wittmack), maize (*Zea mays* L.), and sorghum (*Sorghum bicolor* L.) can be influenced by plant height [18, 29]. However, the relationship between biomass and plant height in orchardgrass remains unclear. To investigate this, we analyzed the relationship between plant height and biomass yield in a GWAS population and found that plant height is an important factor influencing biomass in orchardgrass (Fig. S3). Additionally, plant heights of 90–110 cm contribute to maximum biomass yield in orchardgrass (Fig. 1). These findings suggest that optimal plant height is beneficial for maximizing biomass yield. While most previous studies have focused on the relationship between grain yield and plant height, aboveground biomass is the primary yield source for forage crops. Therefore, our study helps clarify the relationship between plant height and biomass in forage species. Orchardgrass is also a relative of major crops such as rice, barley, and wheat [28], making it valuable for diversifying plant height research in major crops, such as rice and barley. Exploring genes controlling plant height in orchardgrass can further enhance our understanding and improve biomass yield. However, no genes controlling plant height in orchardgrass have been reported thus far.

In our study, we associated plant height with aboveground biomass and concluded that maximum biomass was obtained at around 90 cm plant height in orchardgrass (Fig. 1). We then analyzed plant height in a GWAS population, which varied from 22.88 cm to 160.10 cm (Fig. 2). The abundant phenotypic variation in plant height facilitated the identification of suitable genes controlling it. Our study identified 23 loci and 62 associated genes through GWAS analysis, but no homologous genes regulating plant height were found among these 62 candidate genes. Most of the reported regulatory genes for plant height have focused on domesticated annual crops [17, 18, 58]. However, most orchardgrass used in this study did not undergo artificial domestication, which may explain the absence of homologous genes in our results. Additionally, plant height is controlled by multiple genes, and regulatory genes may differ among species [17], which also accounts for our findings. Given that most plant height genes are associated with phytohormones, *DG2C02300*, the only gene among the 62 candidates involved in plant hormone signal transduction and auxin response, was selected for further investigation (Fig. 3). Plant height is regulated by several phytohormones, such as IAA, GAs, and BRs [2–4]. DG2C02300 belongs to the SAUR gene family and is a homolog of SAUR71 in *Aegilops tauschii*, *Brachypodium distachyon*, *Sorghum bicolor*, and *Zea mays* (Fig. 4). Therefore, we named it *DgSAUR71*. The SAUR gene family, one of three related to early auxin responses, plays a key role in plant growth and development [59–61]. *OsSAUR39* regulates plant growth and development as a negative regulator of auxin synthesis and transport in rice [62]. *AtSAUR63* promotes hypocotyl and stamen filament elongation in *Arabidopsis* [63], and the SAUR50-like protein is involved in heliotropic movements in the common sunflower (*Helianthus annuus*) [64]. Therefore, *DgSAUR71* may regulate plant height by influencing growth and development.

Due to the unstable transformation system of orchardgrass and its close relation to rice, rice serves as a good model plant for orchardgrass research [28]. Therefore, we overexpressed *DgSAUR71* in rice (ZH11) to study its effect on plant height. The plant height of the *DgSAUR71-OE1* line was 14.6 cm shorter than ZH11, while the *DgSAUR71-OE2* line was 7.7 cm shorter (Fig. 5A, B). Overall, the plant height of the overexpressed lines decreased by over 6.7% compared to ZH11. The expression level of *DgSAUR71* in the overexpression lines was significantly higher than in wildtype (Fig. 5C). These results indicate that *DgSAUR71* overexpression slightly reduced plant height in rice, suggesting a negative correlation with plant height. *DgSAUR71* is a candidate gene for controlling plant height in various cereal crops, using rice as a model. Additionally, these results indicated that *DgSAUR71* regulates plant height in orchardgrass and may be applied in breeding new orchardgrass varieties. The *DgSAUR71* function also supports the reliability of our GWAS results. Since plant height in orchardgrass can be controlled by polygenes, further studies are needed to explore the function of other candidate genes and their interactions, which will help elucidate the underlying regulatory mechanisms of plant height in orchardgrass.

## Conclusions

In our study, 23 loci and 62 associated genes related to plant height in orchardgrass were identified through GWAS analysis. *DgSAUR71*, a member of the small auxin-up RNA gene family, was used to investigate its effect on plant height. It was found to slightly reduce plant height in rice and was negatively correlated with plant height. Thus, these results suggest that *DgSAUR71* regulates plant height in orchardgrass and provides new insights for future breeding of orchardgrass varieties.

## Electronic supplementary material

Below is the link to the electronic supplementary material.

Supplementary Material 1: Fig. S1. The linkage disequilibrium decay. Fig. S2. PCR results of DgSAUR71 transgenic lines 1-12 in rice. Fig. S3. The correlation of plant height and biomass. Fig. S4. The correlation of plant height between years and locations. Fig. S5. Go term for candidate genes from GWAS. Fig. S6. The multiple alignment of SAUR71 in different species.

Supplementary Material 2: Table S1 The data of plant height in GWAS population.

Supplementary Material 3: Table S2 The data of the correlation between biomass and plant height in GWAS population.

Supplementary Material 4: Table S3 The primers were used in this study.

Supplementary Material 5: Table S4 The information of candidate loci and genes from GWAS.

Supplementary Material 6: Table S5 The GO and KEGG annotation of candidate genes.

Supplementary Material 7: Table S6 The data of plant height in transgenic lines.

## Data Availability

The authors declare that the data supporting the findings of this study are available within the paper and its supplementary information files. Authors are pleased to share analyzed/raw data upon reasonable request.
