# Supplementary material for: Genome-wide association analysis reveals the function of DgSAUR71 in plant height improvement
Source: BMC Plant Biol. 2025 Feb 22;25:240. doi: 10.1186/s12870-025-06246-x (PMC11846171; doi:10.1186/s12870-025-06246-x)
Supplement: Supplementary file 1 — Supplementary Material 1: Fig. S1. The linkage disequilibrium decay. Fig. S2. PCR results of DgSAUR71 transgenic lines 1-12 in rice. Fig. S3. The correlation of plant height and biomass. Fig. S4. The correlation of plant height between years and locations. Fig. S5. Go term for candidate genes from GWAS. Fig. S6. The multiple alignment of SAUR71 in different species. [file 12870_2025_6246_MOESM1_ESM.docx]

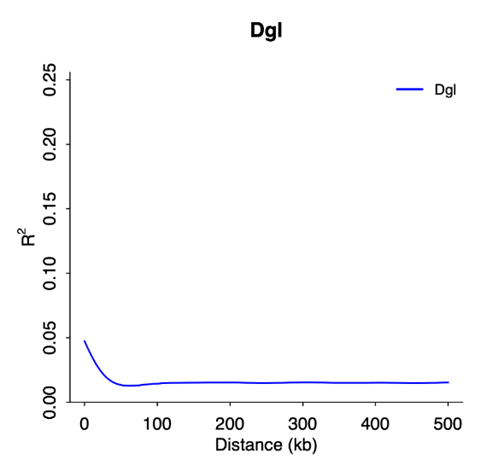


**Fig. S1.** The linkage disequilibrium decay. *p* < 0.05.

**Fig. S2.** PCR results of *DgSAUR71* transgenic lines 1-12 in rice. “M” represents marker, “WT” represents wild type, “C” represents negative control. 12 T_0_ samples was among 12 lanes on the left of marker, among which S1-S5 represents the positive lines.


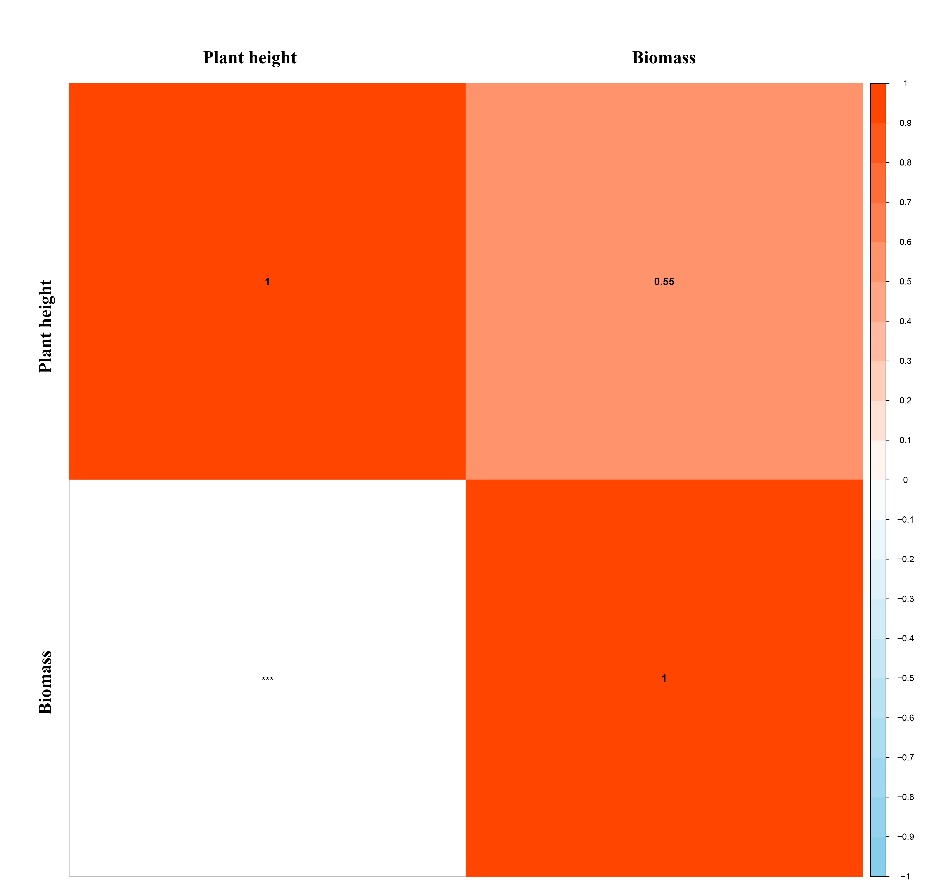


**Fig. S3.** The correlation of plant height and biomass. *** represents *P*<0.001.


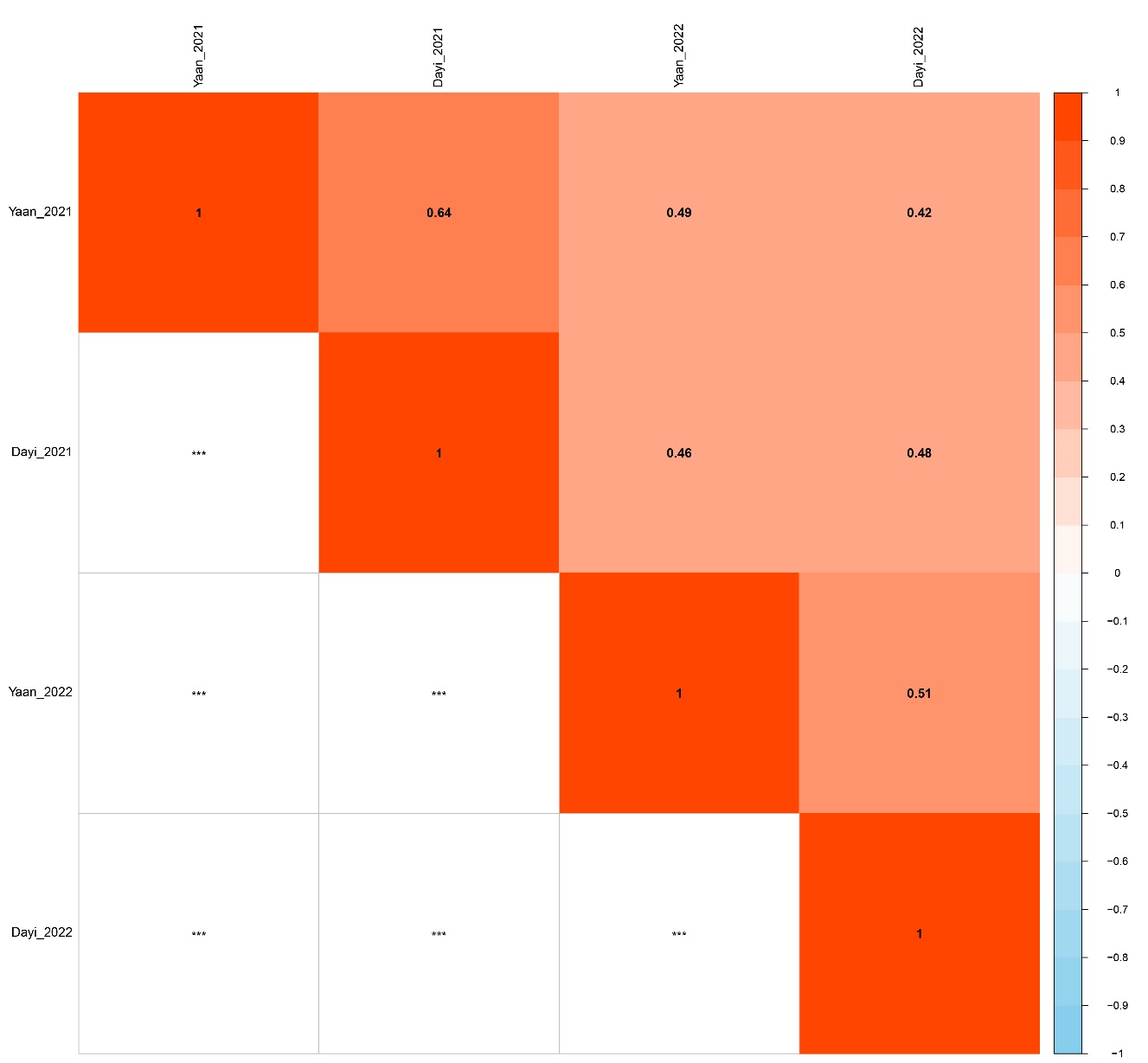


**Fig. S4.** The correlation of plant height between years and environments. *** represents *P*<0.001.


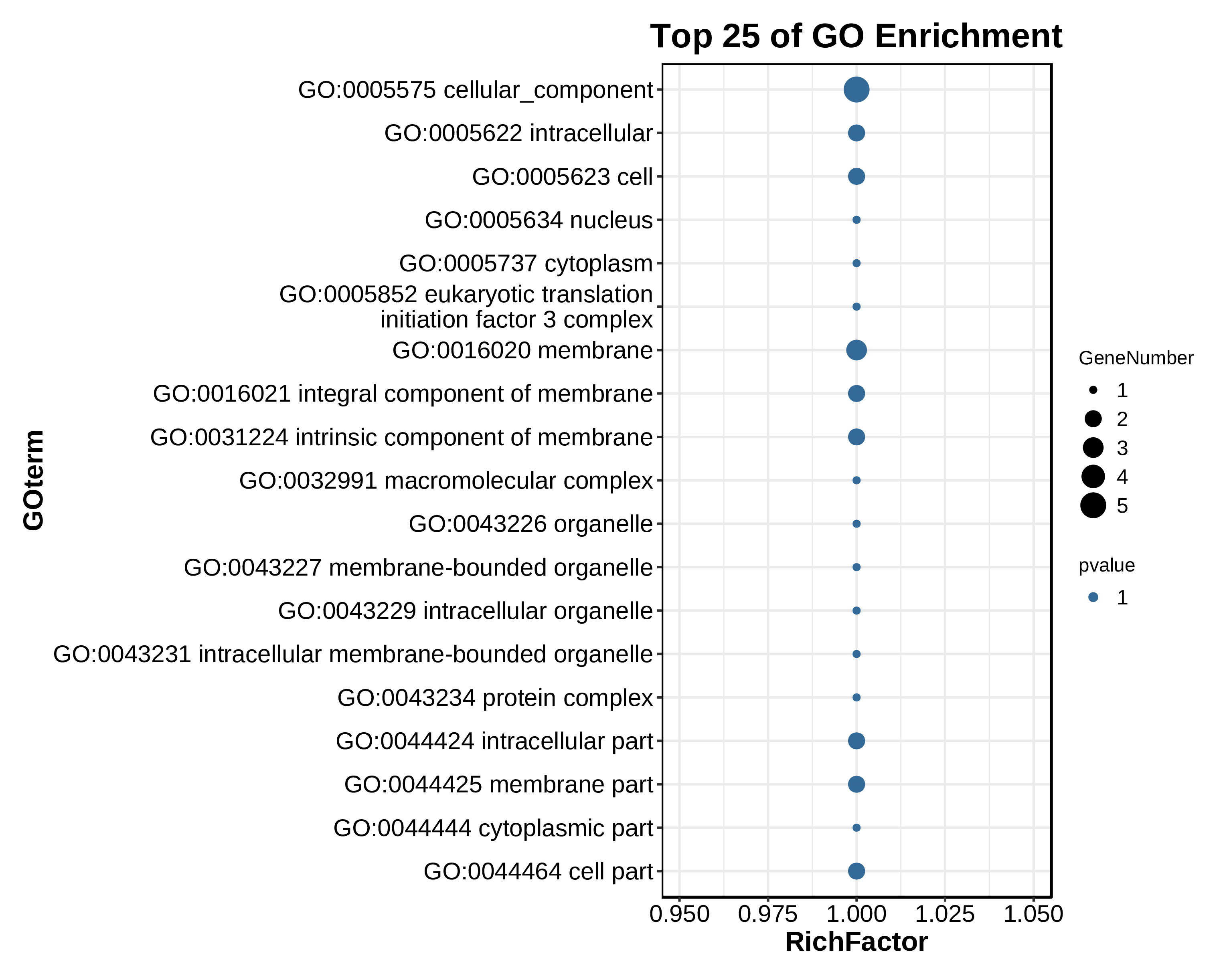

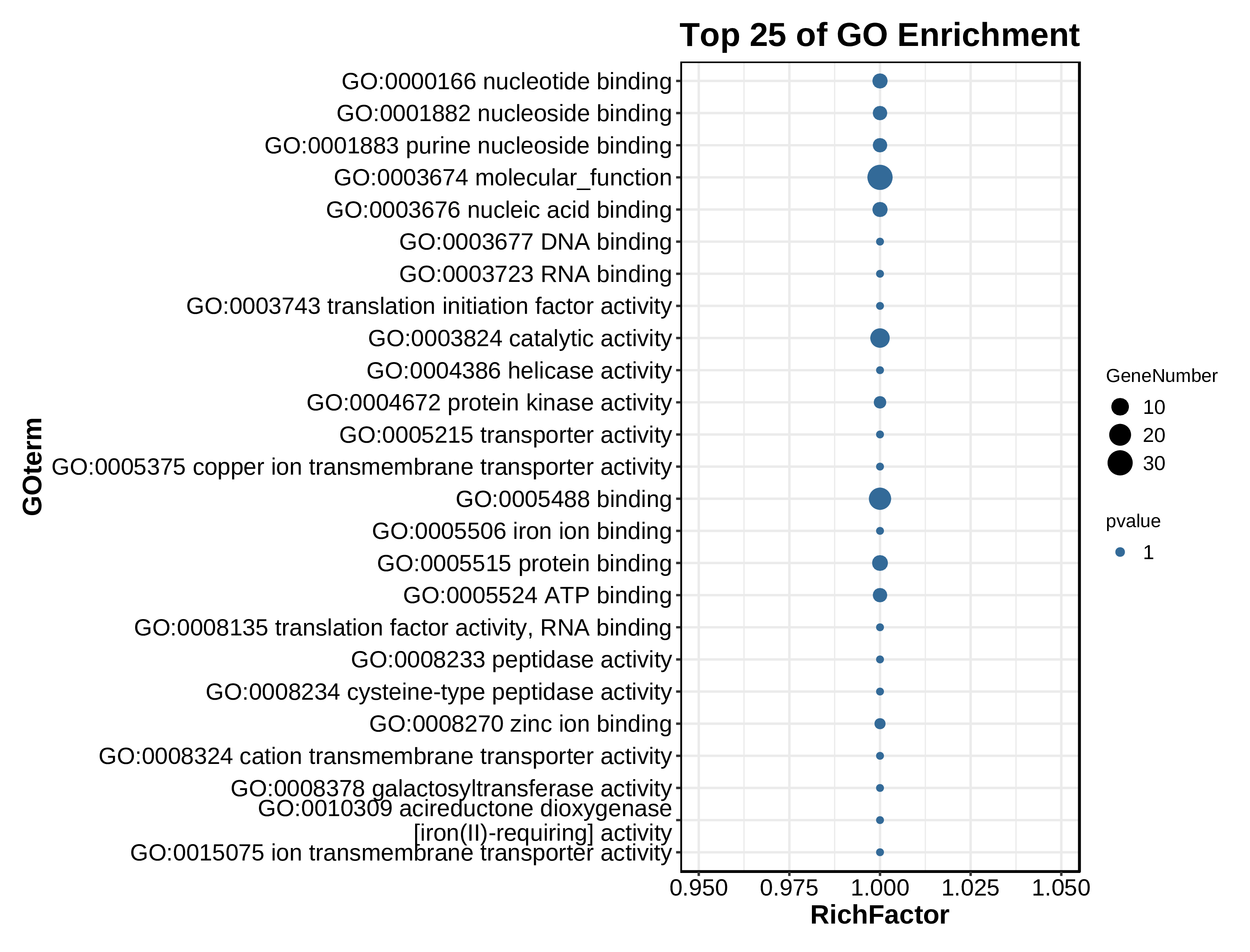


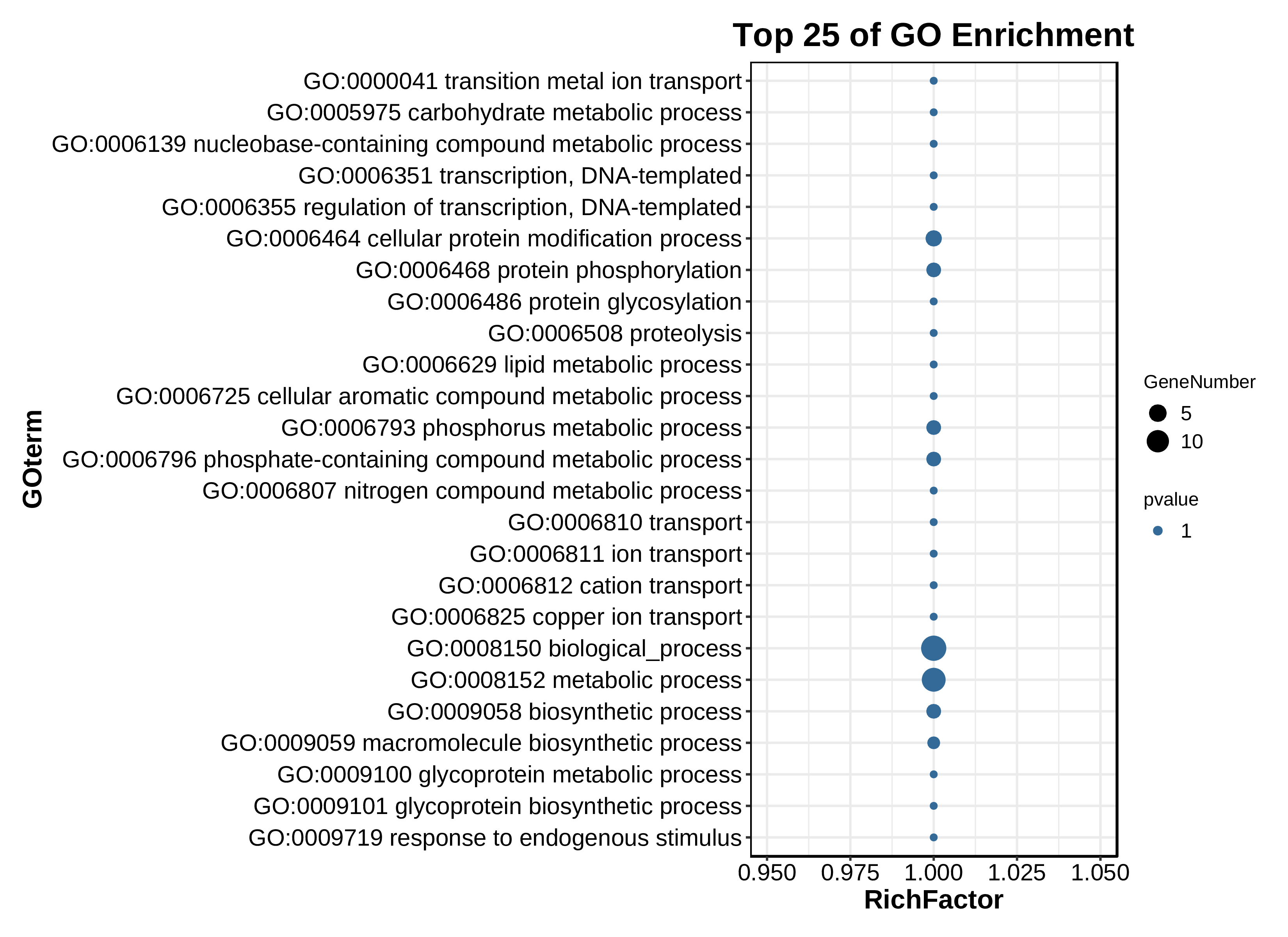


**Fig. S5.** Go term for candidate genes from GWAS.


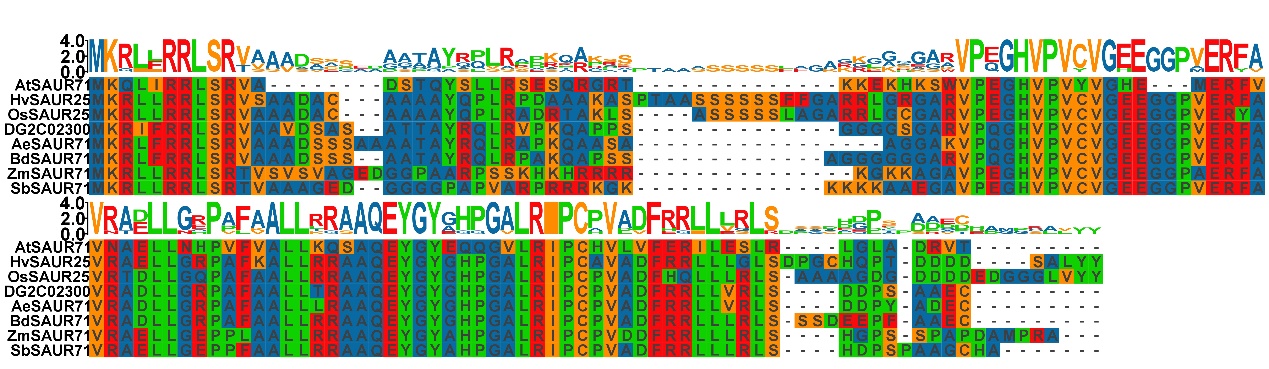


**Fig. S6.** The multiple alignment of SAUR71 in different species.
